# The Use of Polymer Blends in the Treatment of Ocular Diseases

**DOI:** 10.3390/pharmaceutics14071431

**Published:** 2022-07-07

**Authors:** Raquel Gregorio Arribada, Francine Behar-Cohen, Andre Luis Branco de Barros, Armando Silva-Cunha

**Affiliations:** 1 Laboratory of Pharmacotechnics and Pharmaceutical Technology, Faculty of Pharmacy, Federal University of Minas Gerais, Belo Horizonte 31270-901, Brazil; raquelgregorio.ufmg@gmail.com (R.G.A.); albb@ufmg.br (A.L.B.d.B.); armando.cunha.ufmg@gmail.com (A.S.-C.); 2 Inserm UMR_S 1138, Team 17, Physiopathology of Ocular Diseases: Therapeutic Innovations at Centre de Recherche des Cordeliers, 75006 Paris, France; 3 Ophthalmopole at Hôpital Cochin, 75014 Paris, France; 4 Sorbonne Paris Cité, UMR_S 1138, 75006 Paris, France

**Keywords:** polymer blend, biopolymers, ophthalmic administration, drug delivery system, polymer association, ocular use

## Abstract

The eye is an organ with limited drug access due to its anatomical and physiological barriers, and the usual forms of ocular administration are limited in terms of drug penetration, residence time, and bioavailability, as well as low patient compliance. Hence, therapeutic innovations in new drug delivery systems (DDS) have been widely explored since they show numerous advantages over conventional methods, besides delivering the content to the eye without interfering with its normal functioning. Polymers are usually used in DDS and many of them are applicable to ophthalmic use, especially biodegradable ones. Even so, it can be a hard task to find a singular polymer with all the desirable properties to deliver the best performance, and combining two or more polymers in a blend has proven to be more convenient, efficient, and cost-effective. This review was carried out to assess the use of polymer blends as DDS. The search conducted in the databases of Pubmed and Scopus for specific terms revealed that although the physical combination of polymers is largely applied, the term polymer blend still has low compliance.

## 1. Introduction

The eye is an organ with several anatomical and physiological barriers. The anterior segment barriers are designed to keep foreign substances from the environment out, while the blood–retinal barrier is responsible for preventing the entry of the systemic circulation. In this way, delivering drugs to specific intraocular targets at therapeutic levels can be very challenging [1,2,3,4]. Moreover, drug penetration to ocular tissues also depends on the physicochemical properties of the drugs, such as molecular weight, ionic charge, lipophilicity, and aqueous solubility, among others [5].

The treatment of disorders in the anterior segment of the eye can be considered relatively simple because of the easier access to these tissues and consequent patient compliance. Topical instillation, such as solutions, suspensions, and ointments, is the most used approach in this case [6]. Nevertheless, those formulations show very low precorneal residence time, so new technologies are required to enhance drug penetration and bioavailability [7].

The treatment of ocular diseases affecting the posterior segment of the eye, especially the retina, is still a challenge due to the complexity and particularity of the anatomy and physiology of the eye. The conventional routes of administration are found inefficient in delivering drugs to the posterior segment owing to different ocular barriers and limitations of routes. While high dosages of the drug or repeated injections are needed to achieve therapeutic concentrations in the target site, they may also be responsible for toxicity and adverse effects, not only in the eye but in other organs as well [8,9,10].

There has been considerable progress in keeping drug levels constant at the site of action, especially with the development of polymeric drug delivery systems (DDS). The different technologies used in DDS aim for the sustained or prolonged release of the drug at the target tissue. Moreover, an ideal DDS should be easy to administer and deliver the content to the ocular tissue with comfort and without interfering with the vision or normal functioning of the eye [8,11,12,13].

Many polymers are applicable to ophthalmic delivery systems and they are usually classified into natural, synthetic and semisynthetic, which are chemically modified natural polymers [14]. Although several biodegradable polymers have been used in the preclinical stage for the sustained release of drugs to the eye [15], most of the time it is quite challenging finding a polymer with all the desired properties to produce the best DDS, as well as thinking of chemical strategies to overcome this issue.

Therefore, blending two or more polymers is a convenient strategy to form polymeric systems with new and more interesting properties in comparison to the individual components [16]. Among other benefits, this approach may overcome the necessity of synthesizing new monomers and polymerization routes, in such a way that over 30% of commercial polymers in current use are blends [17,18].

Combining polymer characteristics by blending them is one of the most applied strategies to obtain new materials with improved and innovative properties [19], such as impact, crack and aging resistance, and stability at high temperatures [20]. These strategies are also applied to the development of DDS with enhanced performance. Indeed, Changez and collaborators (2003) have demonstrated that a single polymer cannot regulate the drug release rate in a DDS properly, and therefore, the combination of two or more polymers could be a better approach to modulate this property [21].

The microstructure of the blend, that is, the small-scale arrangement of the components, is related to the rheological, optical, and transport properties of the mixture, as well as its thermal and mechanical behavior. It is known that these characteristics can be optimized by controlling the composition of the blend, and even though it is believed that this phenomenon is directly related to the formation of homogeneous or heterogeneous polymer blends, the morphological events involved are still poorly understood [17,22,23].

**Figure 1:**
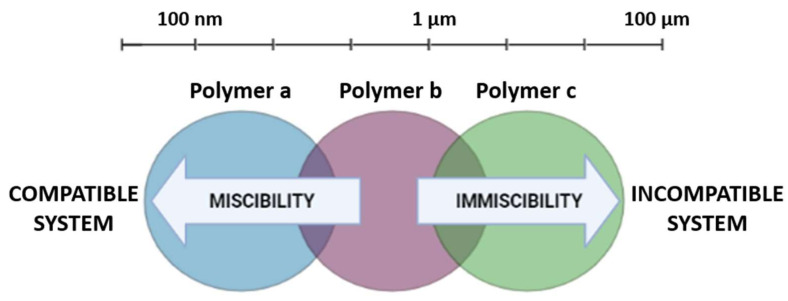
Miscibility vs. compatibility of polymer blends.

Basically, the morphology of the blend is dependent on two factors: the miscibility and compatibility of the polymers used, although their meanings are usually confused. The term miscibility is used to describe the level at which the components in a blend are dispersed into each other and the interactions between them, which are strongly dependent on temperature [24]. According to Imre et al., 2013, “miscibility is a thermodynamic term that describes the behavior of a polymer pair by specifying the number of phases and their composition forming upon blending.” [25]. On the other hand, the term compatibility refers to the final properties of the blend, that is, a blend with improved properties can be considered to be a compatible blend. Therefore, in general, polymers with a high degree of miscibility tend to form homogeneous blends, whereas immiscible polymers form incompatible systems (Figure 1) [26].

Considering these concepts, polymer blends can be distinguished into three different types: completely miscible, partially miscible, and fully immiscible blends [18], knowing that the concept of miscibility is directly related to the energy of the system.

According to thermodynamics, two polymers tend to be completely miscible when the Gibbs free energy of the mixture is negative and the interfacial tension between the components is close to zero, which leads to a uniform single-phase product. This ideal system rarely occurs, though; in fact, most polymers are practically immiscible, in a way that their interactions are considerably high and their entropy very low, making the final properties of the blend weak and with poor mechanical performance [18,27]. Biopolymers are renewable and/or biodegradable polymers, mostly used in the medical field. Biopolymers contain polar groups that can form stronger interactions, especially through induced dipole or dipole–dipole interactions, in a way that the mutual miscibility of the phases is enhanced [25].

Thus, the major problem in blending polymers is to overcome their immiscibility and consequent phase separation. It has been demonstrated that these characteristics are directly related to the morphology of the blend, which plays a key role in dictating the material properties [19,28]. The optimal morphology should consist of small droplets dispersed in a matrix; the main types of morphology that can be observed regarding immiscible polymer blends in the melt state are dispersed, co-continuous, droplet-type, laminar, and fibrillar. Various published studies illustrate that mainly the interfacial tension, the kinematics of the flow, the rheological properties composition, and the elasticity of the components are the key parameters that rule the morphology of the blend. The establishment of each type of morphology depends on the ratio of the blended polymers, as well as their viscosity ratio, interfacial tension, the use of compatibilizers, the addition of a third phase, and processing parameters [27,29].

**Figure 2:**
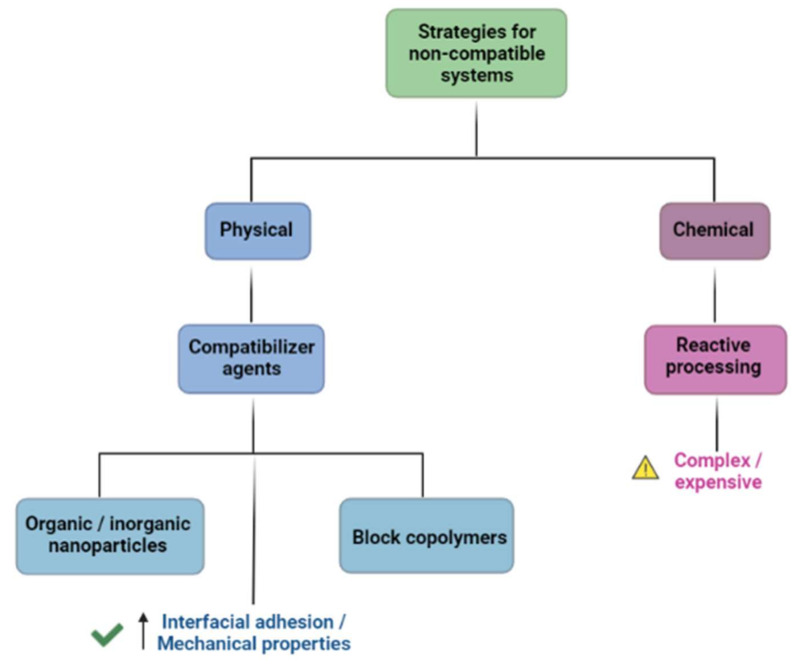
Schematic diagram of some strategies used to circumvent the outcomes of non-compatible systems.

Different strategies can be applied in order to prevent or at least minimize the outcomes of having an immiscible or non-compatible system, and they can be both physical and chemical approaches. Chemical means evolve reactive processing, which can be either more expensive or more complex to perform, while physical approaches include mainly compatibilizer agents. Compatibilizers are macromolecules shown to soften unfavorable contacts among the components of the blend and increase the interfacial adhesion. The use of compatibilizers leads to improved mechanical properties and can be represented by inorganic and organic nanoparticles, as well as block copolymers (Figure 2). These last ones have great commercial and scientific interest due to their special properties; they are species with chains in a blocky structure, with one block miscible with one component of the blend and the second block miscible with the other component, and they can be pre-made or generated in-situ during the blending process [19,24,30,31].

## 2. Methods

This review was performed in the databases Pubmed (https://pubmed.ncbi.nlm.nih.gov/advanced/, accessed on 10 March 2020), and Scopus (https://www.scopus.com/home.uri, accessed on 10 March 2020). Considering that the search for polymer-based works results in over 80,000 published papers, some criteria have been adopted. MeSH terms were used as a search strategy for a more accurate search; since a MeSH term for “polymer blend” (and its derivatives) could not be found, the following MeSH terms and their intra-terms were used: [polymers], [drug delivery systems (delivery system, drug), (drug targeting), (targetings, drug)], [administration, ophthalmic (ophthalmic administration), (administration, ocular), (ocular administrations)]. The research was carried out until April 2021, and a filter for publications from the last 5 years was also applied. Searches have shown a total of 127 papers in Pubmed and 81 papers in Scopus, 51 of which were common to both. All the papers that did not consist of polymer blends were excluded from the results. Table 1 contains the list of all papers regarding polymer blends found in the two searches (14 papers in the full-text format could not be found, so they were not included on this list).

## 3. Results

Once all the available papers were collected, the first task was to determine which ones used, in fact, a polymer blend in their work, which corresponds to the first column of Table 1. We found that basically only half of the papers listed in the table used blends in the formulations, while the other half either used only one polymer or no polymer at all (even though the search was specific for polymeric materials, many papers were about other types of technology, such as lipidic formulations).

Based on this search using specific MeSH terms, and among the works that fit the proposal of this paper, most of them present new technologies simply as DDS for ocular use, so as to improve residence time and control drug release, while others offer new formulations for specific targets. Herein, we discuss and show some examples of the most popular polymers in ocular use, as well as target tissues and/or diseases found in this search; some other papers were also included as additional information.

### 3.1. Common Polymers in Ocular Use

Nowadays, various synthetic and natural polymers are being used in DDS, either individually or combined in blends. Because they have different physicochemical properties and different ways of interacting with cells, some polymers are better suited for certain targets, depending on the tissue characteristics and the aimed type of drug release. In Table 2, we present a summary of the most-used polymers and DDS for the most commonly discussed diseases and/or tissues. We also present a brief explanation of the most important characteristics of each polymer.

#### 3.1.1. Synthetic Polymers

Synthetic polymers have been widely used in DDS. Despite the challenge of producing biocompatible, non-toxic materials, synthesizing them enables the modulation of specific properties and, consequently, the final performance of the material.

Poly lactic-co-glycolic acid (PLGA) was the first polymer approved by the Food and Drug Administration (FDA) for DDS and is still largely used due to its properties. PLGA is a synthetic hydrophobic polymer made from the copolymers PLA and PGA, which has emerged as an important non-toxic, biodegradable, and biocompatible material [110]. One of the great features of PLGA is the possibility of modulating mechanical strength, swelling behavior, drug release, and degradation rate, among others. These features can be achieved by varying the proportion between the two copolymers, PLA being more hydrophobic and PGA more hydrophilic [111,112].

Polycaprolactone (PCL) is a semi-crystalline polyester, also approved by the FDA for biomedical applications, which presents great biocompatibility and low toxicity [113]. PCL has good thermal stability and behaves as a flexible plastic at room temperature, which gives it the ability to mold into different forms and its surface can be easily modified; even so, PCL has relatively poor mechanical properties, and that is why it is often combined with modifiers and/or blended with other polymers [114,115,116].

Polyethylene glycol (PEG) is a hydrophilic, neutral polymer of ethylene oxide widely used as a coating of nanoparticles (pegylation). This is a strategy to increase the lifetime of the drug carrier or to give a hydrophilic character to hydrophobic systems [117,118]. PEG is commercially available with different degrees of polymerization and its commercial fame comes from its flexibility, amphiphilicity, hydration capacity, and biocompatibility [119].

Hydroxypropyl methylcellulose (HPMC) is a cellulose non-ionic derivative that, unlike cellulose, is soluble in water and many other organic solvents. Because of its hydrophilic (hydroxy) and hydrophobic (ether) groups, this material shows excellent drug-polymer miscibility. The HPMC property of swelling when in contact with water creates a gel layer that modulates drug release and enhances bioadhesion, which makes HPMC an excellent base for hydrogels and mucoadhesive nanoparticles [120,121,122].

Carbopols, or carbomers, are synthetic polymers derived from acrylic acid, of high molecular weight and anionic character. Besides being well-tolerated and non-toxic, their rheological properties are remarkable. When dispersed in water, carbopols present viscoelastic behavior, and after suffering the neutralization of their acid groups with an inorganic base, they undergo a sol-gel transition due to the cross-linking of their polymer chains [123,124].

Eudragit^®^ is the commercial name for different types of polymethacrylates copolymers. It is presented in different forms, the most used in DDS being the neutral types Eudragit RL and Eudragit RS (pH-independent solubility) as coating materials to help control drug release [125].

Polyvinyl alcohol (PVA) and Polyvinylpyrrolidone (PVP) are thermoplastic, non-toxic, biocompatible, and water-soluble polymers with tunable properties [126,127]. These two compounds are often blended to overcome their individual limitations and can be extensively found in ocular DDS as contact lenses and hydrogels [128,129,130].

Poloxamers (Pluronic^®^, Kolliphor^®^…) consist of nonionic triblock copolymers composed of hydrophobic polyoxypropylene and hydrophilic polyoxyethylene chains, with a thermosensitive characteristic. Poloxamers suffer a sol-gel transition when reaching physiological temperatures and this is why they are known as in situ forming gels [131,132].

#### 3.1.2. Natural Polymers

The use of natural polymers, or biopolymers, is highly targeted due to the lower toxicity and biocompatibility that these compounds present and, most importantly, because of their abundance in nature. Nevertheless, it can be quite challenging to find an already existing polymer with the desired characteristics for a DDS. That is why natural polymers are commonly blended with synthetic polymers or go through surface modification.

Chitosan is a cationic polysaccharide derived from chitin and is thus biodegradable and biocompatible. In addition to its usage as a matrix for DDS, chitosan itself shows antimicrobial, antifungal, and wound-healing features due to its polycationic structure. Despite being water-insoluble, the chitosan structure can also be modified without changing its physicochemical and biochemical properties, which gives this polymer great versatility [81].

Hyaluronic acid (HA) is an anionic glycosaminoglycan composed of D-glucuronic acid and N-acetyl glucosamine groups, with several roles as the major extracellular matrix component. HA has good anti-inflammatory and cell permeability properties. Moreover, its high moisture retention allows the formation of a network of hydrogen bonds, leading to the easy formation of a hydrogel with great viscoelastic characteristics, making this polymer an excellent candidate for DDS [133,134,135].

Gelatin is a protein obtained from the denaturation of collagen. Gelatin differs from other polymers because of the presence of amino acid sequences in its structure, which play an important role in modulating cell adhesion [136]. Gelatin has both cationic and anionic groups and an amphiphilic structure, making it water-soluble and widely used as a hydrogel matrix. In addition, the use of cross-linkers and targeting ligands to modify its functional groups enhances the interest in this biopolymer [137,138].

Inulin is a natural fructan polysaccharide that can be found in a variety of plants. This compound is made of fructose and glucose units in a flexible backbone, which gives it considerable versatility [139]. Inulin has been widely used in drug delivery systems because of its rapid water solubility, low friability, and stability via oral administration [140], as well as a successful ocular permeation enhancer [141].

### 3.2. DDS Made from Polymer Blends

#### 3.2.1. Hydrogels

In situ-forming gels and hydrogels are the most-used technology as DDS for general ocular use. Hydrogels are aqueous formulations composed of three-dimensional polymer networks, with a structure similar to the extracellular matrix. Their viscosity helps increase residence time and can be achieved by using polymers that undergo sol-gel transition when in contact with the ocular surface [45,90,93]. The sol-gel transition can be triggered by external stimuli such as temperature, pH, and ionic strength, for instance. Other strategies may still be used to enhance the gelling property, such as adding different salts and viscosity-enhancing agents (PEG, PVA…) [87,99], and also to sustain drug release by employing, for example, nanoparticles as the drug carrier within the gel [53]. Different polymers, including carbopol, PEG, HA, and HPMC, are very popular as hydrophilic matrices, but the most common ones found are the poloxamers (Pluronic^®^).

#### 3.2.2. Nano and Microparticulated Systems

Although hydrogels present numerous advantages, especially prolonged residence time, they are not compatible with hydrophobic drugs due to the highwater content of the formulation. Therefore, nanoparticulated systems can be a good strategy to carry lipophilic drugs [106]. Yousry and collaborators (2017) developed Eudragit^®^RS100 with either PLGA or PCL blends to carry vancomycin [98]. At first, Eudragit^®^RS100 was added to provide a positive charge to the particles in order to increase its residence in the ocular tissues, but after the drug encapsulation efficiency (EE%) test, it was noticed that adding Eudragit^®^ to the PCL formulation enhanced the EE% because of its quaternary ammonium groups that interacted with the drug. In another study, also with PCL, the authors proposed freeze-dried polymeric nanoparticles for ocular use. In this case, PCL was blended with Pluronic^®^188, which worked as a stabilizer, and PEG. The last was shown to be a great cryoprotectant agent, and the final result was a non-collapsed freeze-dried matrix [100].

Polymer blends can also be used to form nanoparticulated systems with enhanced bioadhesion and permeation. Di prima et al., 2019 prepared PEGylated inulin-based self-assembling nanoparticles to carry corticosteroids. According to the authors, PEG chains can promote hydrogen bonds between polymers and mucins on the ocular surface, which increases drug permeation. In this study, the in vitro transwell study demonstrated the ability of the carriers to cross not only the corneal epithelium but also the cellular monolayer. Moreover, ex vivo transcorneal permeation studies showed the enhanced penetration and permeation of the tested drugs (dexamethasone, triamcinolone, and triamcinolone acetonide) in the PEGylated nanoparticles compared to the non-PEGylated ones [142].

Another group proposed an inulin-based DDS to form nanosystems, but, on the contrary, they synthesized an inulin–PLA amphiphilic copolymer, using different PLA molecular weights, self-assembled into nanoparticles. It was observed that the water dispersibility of the system was dependent on PLA molecular weight. Moreover, both nanoprecipitation and film rehydration methods were evaluated. In the rehydration method, the copolymer tends to form bigger particles and vesicles, in the microscale form, while nanoprecipitation provided nanoaggregate forms [143].

Microparticulated systems are also an intelligent approach to enclosing different molecules. Even though micro and nanoparticles share several advantages in sustained drug release, the size difference may cause variations in drug-loading efficiency, permeability, and cell entry, for example [144,145]. Khan and colleagues (2017) worked on mucoadhesive chitosan microparticles containing tobramycin sulfate, an aminoglycoside antibiotic. These sterilized microparticles were then dispersed into a poloxamer 407 solution with chitosan HCL to form the in situ gel. The authors reported that the pH of the chitosan solution had a direct impact on microparticle formation, in a way that microparticles could not be formed when the chitosan solution was acid (pH < 4.5). Moreover, microparticle size and entrapment efficiency increased with higher drug and chitosan concentration, the first being due to greater accessibility of the drug and the latter because of high crosslink density. The association of poloxamer and chitosan to form the in situ gel resulted in a biomaterial with better mechanical strength and prolonged residence time on the ocular surface [60]. Another group aimed to encapsulate sorafenib tosylate, a drug used in the treatment of retinopathies and also in mucoadhesive microparticles. In this study, chitosan was functionalized with _L_-arginine, resulting in an amphiphilic copolymer. Microparticles were obtained by the coacervation phase separation technique and in vitro studies showed strong time- and concentration-dependent adhesive interaction between the DDS and mucin. The in vitro permeation assay demonstrated a superior cumulative amount of drug permeated from the loaded microparticles compared to the drug solution [146]. Besides topical application, microparticles can also be administered by other routes. In an interesting assay conducted by Xia and collaborators (2020), PLGA microparticles intended for subconjunctival injection were physically blended with HA and methylcellulose. This strategy was considered because of the shear-thinning properties of HA and methylcellulose that would facilitate the microparticles’ injection through fine-gauged needles and modulate the drug release kinetics of the drug. They have observed that the presence of the microparticles enhanced the viscosity of the HA–methylcellulose solution without interfering with its shear-thinning properties. Moreover, the blended polymers could prevent microparticle leakage through the needle puncture at the site of injection by up to 80% for 30 days, and drug release lasted over 3 weeks. According to the authors, this system represents a promising direction for successful injections of microparticles without formulation loss or needle clogging [34].

#### 3.2.3. Polymeric Micelles

Polymeric micelles are another type of nanostructured particle based on amphiphilic molecules or block copolymers with the ability to self-assemble into organized systems in aqueous media [95]. In a work published by Liu et al., 2019, they developed stable micelles based on a self-assembly NH_2_-PEG-*b*-PLA deblock copolymer, and HPMC was selected as the stabilizer and viscosity-improving agent. The characterization tests showed that HPMC could improve the stability of the formulation at room temperature because of the methoxy and hydroxyl groups of this polymer, capable of forming hydrogen bonds with NH_2_-PEG. The authors also attribute an improved retention ability and lower surface tension to the mucoadhesive nature of HPMC [40]. Chitosan is also used as a superficial coat for non-mucoadhesive polymers, and due to its cationic profile, it can help to open the tight junctions of the corneal epithelium, which improves the performance of the micelles [79]. A self-assembling, multi-layered nanomicellar formulation was also the choice of Mandal and collaborators (2019) to vehicle small peptides for topical use. Despite PLGA being the most-used polymer so far in DDS, they reported that the residues from PLGA can degrade lysine residues of peptides and, therefore, they came up with a blend of polyoxyethylene hydrogenated castor oil 40 (HCO-40) and octoxynol 40 (OC-40). The combination of both polymers demonstrated the highest entrapment and loading efficiency, as well as better thermodynamic stability. According to the authors, adding a second polymer can generate very stable systems, up to 65 °C, due to the strong hydrogen bonds formed, besides controlling the release behavior. A nanomicellar formulation using these polymers combined has already been used for small hydrophobic molecules and completed phase 3 of clinical trials with promising results for dry eye disease [48,147].

Inulin was the choice of Rassu et al., 2021, to form polymeric micelles conjugated to α-tocopherol by ester conjugation. This system aimed to incorporate hydrophobic drugs, such as curcumin, to treat diabetic retinopathy and other retinal diseases. About 16 nm micelles were obtained, which were tested in transport studies in HRPE cell monolayers. The authors have observed that these monolayers can induce an active efflux of not only free curcumin but also the loaded micelles; however, this system was able to protect curcumin in the aqueous media and control its release, which prolonged the ability of curcumin to protect the tight junctions of retinal pigment epithelium cells. The authors concluded that this inulin system is suitable for intraocular administration to treat diabetic eye diseases [148].

After having developed an inulin-based DDS to form nanoparticles [142], Di Prima and collaborators (2021) used the same amphiphilic self-assembling polymers to form micelles containing dexamethasone, which were functionalized with ocular permeation enhancers in this study. Taurine, carnitine, and creatine were tested because of their ability to interact with specific transporters located on the corneal surface. The group has observed that the different functionalizations had led to different mucoadhesive properties and that the ex vivo permeation assay using Franz cells and bovine corneas also revealed different permeation efficacy and entrapment ability, PEG_2000_ being the best permeation enhancer and creatine the least effective enhancer molecule for both permeation and entrapment ability [141].

OTX-101 (Cequa^TM^; Sun Pharmaceutical Industries, Inc., Cranbury, NJ, USA) is an FDA-approved nanomicellar aqueous solution containing cyclosporine, for the treatment of dry eye disease. During the preclinical assays, the authors used New Zealand rabbits to evaluate the ocular distribution of cyclosporine after topical instillation. Tests pointed out that this is a well-tolerated product, even after multiple instillations, with high cyclosporine levels throughout the anterior chamber, and minimal systemic exposure compared to the control group [149,150]. Because of these great results, the OTX-101 product proceeded to a phase 3 clinical trial. This study sought to assess the safety and efficacy of the nanomicellar solution in patients with dry eye syndrome. Clinically and statistically significant improvements could be observed in the treated patients after 28 days of treatment initiation compared to vehicle groups, which favors the use of this system as a treatment for dry eye [151].

#### 3.2.4. Ocular Inserts

One of the most studied technologies for ocular devices as DDS is ocular inserts, which are solid or semi-solid devices, usually made of polymeric materials. These inserts should be translucent, smooth in texture, and uniform without imperfections [152]. There are some ocular implants already approved by the regulatory agencies. For the posterior segment of the eye, there are Retisert, Iluvien, and Yutiq, which are not biodegradable, and Ozurdex and Durysta, which are biodegradable implants made of PLGA to carry dexamethasone and brimonidine, respectively [153,154,155]. Although blends represent an excellent alternative, more studies aiming to know in detail the behavior of these materials in ocular tissues are needed.

According to Jain and collaborators (2011), inserts provide accurate dosing, no requirement of additives, and a reduction in systemic absorption, among others [156]. In this study, PVA and gelatin were blended to form a system with superior mechanical properties, due to PVA, and improved mucoadhesiveness, due to gelatin, for the topical ocular administration of ciprofloxacin hydrochloride. Glycerol was used as a nontoxic plasticizer, which provided elasticity to the inserts. The ocular inserts were prepared by esterification of the hydroxyl group of PVA with the carboxyl group of gelatine and their performances were evaluated in vitro, in vivo, and ex vivo. The resulting implants had great transparency and smooth and soft surfaces upon hydration, making them comfortable in the eye. Besides that, they showed in vitro drug release over 24 h, greater dye penetration in deeper ocular tissues of goat eyeballs, and also no signs of toxicity by in vitro and in vivo tests in albino rabbits.

In a study conducted in 2019, microfibrillar polymeric ocular inserts were fabricated for triamcinolone acetonide delivery and, for that, poly (1,4-butylene succinate), extended with 1,6-diisocyanatohexane (PBS), was electrospun into scaffolds. Because of the low wettability presented by PBS, different biopolymers (inulin, heparin, and α,β-poly (N-2-hydroxyethyl)-D,L-aspartamide (PHEA)) were conjugated to the surface of the scaffolds in order to improve its mucoadhesiveness and become a suitable material for an ocular insert. The characterization tests showed that the functionalized scaffolds promoted a strong and stable connection between the inserts and mucin, and consequently, an improved residence time, as expected. Besides the loading efficiency, drug absorption via transcorneal permeation also showed better results compared to the pure PBS scaffolds, being a potential self-administrable and efficient ocular insert [47].

In another similar study to produce biodegradable ocular inserts by the film-casting method for the delivery of brimonidine tartarate, PVP K-90 was used as an insert-forming polymer and different hydrophilic polymers were tested as bioadhesive materials. According to the results, the authors explain that drug release depends on the water accessibility into the matrix of the implant, which is directly related to its capacity for swelling. It means that the hydrophilic polymer breaks the polymer–polymer bonds and creates water–polymer bonds. The more hydrophilic the polymer is, such as HPMC and chitosan, the weaker the hydrogen bonding within, which leads to the rapid penetration of water and consequently rapid drug release. It is well known, though, that the capacity of the swelling of the polymer is essential for its bioadhesiveness. Therefore, the choice of the polymer should be well evaluated so as to modulate the bioadhesive potential of the insert and also the content release [152].

### 3.3. Application of Polymer Blends in Various Ocular Conditions

#### 3.3.1. Glaucoma

Glaucoma is a frequent and blinding eye disease, often associated with increased intraocular pressure (IOP) that may evolve to changes in the optic nerve and subsequent visual field loss. Glaucoma therapies consist primarily of topical eye drops containing drugs such as timolol maleate, brinzolamide, or prostaglandin analogs and combinations [157,158,159]. One of the major challenges in glaucoma treatment is patient compliance, which can be improved by reducing the frequency of instillation. Thus, formulations that enhance the residence time and subsequently increase ocular drug levels and release duration are of great interest. Polymeric nanoparticles and hydrogels are popular systems when trying to develop new formulations to treat glaucoma. A common strategy is to coat the polymeric particle with a different polymer to enhance the performance of the system.

In 2020, Shahab and coworkers proposed mucoadhesive nanoparticles made of PCL, coated with chitosan and PVA, to carry dorzolamide. Based on a 3^3^ Box-Behnken design, they observed that the association was positive in terms of performance; while an increase in the concentration of chitosan and PCL led to bigger particles and, consequently, a slower drug release, PVA enhanced the system stability, led to a decrease in particle size, and an increase in drug release. Chitosan also augmented almost 50% of the mucoadhesion on pig eyes. It is worth mentioning that the paper’s title cites only the use of PLC as the main polymer and chitosan as the coating material; but, in fact, PVA is used with twice the amount of chitosan [35]. Another way of improving the performance of chitosan nanoparticles is by PEGylating chitosan using an ionic gelation method with sodium tripolyphosphate (TPP), which happens spontaneously when preparing the formulation. The higher the PEG molecular weight and concentration, the higher the particle size and polydispersity index; on the other hand, the negative charge of PEG balances the positive charge of chitosan, improving the biocompatibility of the nanoparticles. One should take into account, though, that PEG can compete with the drug during the ionic gelation method of chitosan and can, therefore, decrease the encapsulation efficiency if too concentrated. In this study, the authors also verified that drug transcorneal permeation was considerably higher for the PEG-modified nanoparticles in comparison to chitosan nanoparticles and drug dispersion. They also pointed out that coating chitosan nanoparticles with PEG does not enhance the interaction with the corneal epithelium, but makes it easier for the transcellular transport of the drug [66].

Some authors have investigated the use of polymeric transparent composite films loaded with timolol to overcome, for example, the dry eye resulting from glaucoma using HA. Tighsazzadeh et al. have reported that HA and HPMC can individually produce optimum ocular films, but the association of both in a composite enlarges the performance of the system by combining the swelling capacity of HA with the film-forming ability of HPMC [39]. In another study, in which HA was used for its bioadhesiveness and biocompatibility, the authors managed to incorporate acetazolamide, a poorly soluble drug, into cyclodextrins made of the natural oligosaccharide HPβCD; this complex was then incorporated into an HA film during cross-linking with PEG. The resulting gel films proved to be smooth, biocompatible, bioadhesive, and with good mechanical characteristics. In the in vivo tests, the films were adhered to the bulbar conjunctiva and showed a great intraocular pressure decrease in normotensive male rabbits, which lasted for almost 20 h [61].

No doubt, when it comes to glaucoma treatment, the most studied technology is in situ hydrogels, with gelatin being the major choice to compose the matrix of these systems. El-Feky and co-workers (2018) have assessed the feasibility of the topical delivery of timolol within chitosan–gelatin crosslinked with oxidized sucrose hydrogel, obtaining good performance results. In contrast, Chou and collaborators (2016) studied a gelatin–poly (N-isopropylacrylamide) in situ gel containing pilocarpine. They demonstrated that the gel strength of the gelatin (also called bloom index) has a direct influence on the mechanical, rheological, and thermal properties of the polymer, besides controlling biodegradation and drug release mechanisms. Results suggest that increasing the bloom value of gelatin leads to a slower degradation rate of the system due to triple-helix contents formed in the structure, as well as an increased drug encapsulation. Besides that, the intracameral injection in rabbits’ eyes revealed a continuous decrease in the intraocular pressure (IOP) and pupil diameter for over 14 days [71]. Instead of using gelatin, another group came up with pH-triggered HPMC-carbopol hydrogels containing dorzolamide, that showed pseudoplastic behavior and appropriate gel strength in physiological conditions [43].

Ng and collaborators (2015) proposed the use of timolol on a polymer blend microfilm, placed in the subconjunctiva of non-human primates, for treating glaucoma. To prevent burst release, they blended PCL and PEG copolymers (80:20 PCL:PEG in a sandwich film (blank layer–drug layer–blank layer), and they achieved 2.7 µg of daily timolol in vitro release for 3 months in a zero-order release. The in vivo analysis demonstrated the statistical difference of the blended film in reducing the IOP over time compared to the topical administration, as well as great biocompatibility and no dislocation of the microfilm in the eye. It is important to notice that the in vivo effects (~5 months) lasted longer than the in vitro release, probably due to slower subconjunctival clearance (NG et al., 2015).

Another approach to treating glaucoma was assessed by Natu et al., 2011. They proposed an implant made of a blend of PCL and lutrol F127, containing dorzolamide hydrochloride, to be placed in the subconjunctival space of rabbits. As has also been justified in other studies, PCL was used because of its biocompatibility and slow degradation rate, while lutrol was used as a release modulator (NATU et al., 2011).

In another study, the researchers proposed the fabrication of a multifunctional polymeric microstent for suprachoroidal drainage. The goal was to overcome the inherited and undesired fibrosis, hypotony, and damage to adjacent tissues associated with the use of non-degradable glaucoma implants. In this case, a blend of PCL with poly ((ε-caprolactone)-*co*-glycolide) (PCG) was used as a base to prepare the microstents. This semi-crystalline matrix combines the good mechanical properties of PCL with the tailored degradation pattern of PCG, as well as proper thermal stability. Even so, steam, heat, and ethylene oxide sterilization were unsuitable, in a way that only frozen samples in dry ice prior to γ-sterilization did not suffer the degradation or modification of the mechanical properties. Though mild inflammation was observed after the microstent implantation in rabbit eyes, it could be well controlled and disappeared within a few days [159].

#### 3.3.2. Dry Eye Syndrome

Luo and collaborators (2017) assessed the applicability of a system to treat dry eye that had been previously developed by the same group to carry pilocarpine, an antiglaucoma drug. In this earlier study, the intraocular administration of pilocarpine in a gelatin-*g*-poly (N-isopropylacrylamide) carrier led to better bioavailability and extended pharmacological response compared to the intracameral injection of the free drug. According to the authors, the outstanding adhesion capacity of gelatin relies on its viscosity-building effects, which is why grafting it with thermo-responsive polymers can produce great in situ forming DDS. With this in mind, they came up with the idea of using this system for the management of dry eye symptoms. They used this in situ gel to vehicle epigallocatechin gallate to treat dry eye symptoms in a rabbit model, which showed limited disease progression, cellular inflammation, and oxidative stress [68].

Another group of researchers also studied an in situ gel to treat dry eye, but in this case, using HPMC E-15 and sodium alginate as a matrix to carry levofloxacin. Sodium alginate is a gelling agent that forms low viscosity liquids due to its high content of glucuronic acid, and HPMC was used to adjust the viscosity of the gel. The liquid formulation transformed itself into a gel when in contact with the ocular surface (pH 7.4) and the better corneal permeation compared to levofloxacin eye drops concerns the bioadhesion property of HPMC [44]. On the other hand, Yu et al. investigated the use of block polymer mPEG-PLA micelles for treating dry eye, lyophilized to enhance the stability of the formulation using mPEG2000 as the stabilizer. Particle size and encapsulation efficiency were dependent on the mPEG/PLA ratio; the higher the concentration of PEG, the higher the particle size and encapsulation of cyclosporine A. The ocular distribution study demonstrated that the maximum concentration of cyclosporine after the instillation of the loaded micelles was 4.5-fold higher compared to the administration of a cyclosporine emulsion. Besides that, the formulation was able to sustain cyclosporin concentration in tear fluid better than the emulsion [56].

#### 3.3.3. Infectious Keratitis

Infectious keratitis is the inflammation of the cornea caused mostly by bacteria and fungi associated with the extended use of ocular lenses and ocular trauma. This is why infectious keratitis is one of the leading causes of blindness and endophthalmitis worldwide [33,160,161].

In a study to treat bacterial keratitis, the authors proposed freeze-dried solid mucoadhesive matrices made of HPMC and HA as a suitable system to overcome the instability of peptides and enhance precorneal residence time. The formulation showed great physicochemical stability, with no degradation, for up to 6 months and peptide antimicrobial activity for up to 15 months of storage, due to the freeze-drying process and the cryoprotectant agents applied, such as mannitol. Moreover, the entrapment efficiency could be optimized by the addition of trehalose, a sugar capable of changing the polymer gel structure [33]. Similarly, Sebastián-Morelló and co-workers (2017) also used HPMC as the matrix of an ocular insert to vehicle moxifloxacin as a potential treatment for bacterial keratitis. In this study, PVP and PEG were added as bioadhesive materials and, while the concentrations of the drug and PEG were kept constant, the concentrations of HPMC and PVP were tested to get the best formulation. It was observed that the higher the concentration of HPMC and PVP ((PVP) > (HPMC)) the better the consistency of the insert for lamination, which led to a homogeneous matrix without crystallization. Moreover, the formulation presented a great ability to capture water, which is an important characteristic for bioadhesion and content release. In the *ex-vivo* ocular diffusion studies, the authors found that the insert had been transformed into a gel, which prolonged the adhesion to the cornea and enabled better drug permeation compared to current commercial forms [62].

Another strategy was performed by Eid et al., 2019, who assessed the influence of coating solid lipid nanoparticles with chitosan and PEG on the release of ofloxacin for the topical therapy of bacterial keratitis. It was seen that the capacity of increasing drug entrapment by PEGylating the nanoparticles depends on the levels of the lipid in the formulation and also on appropriate particle size. It means that the larger the particle is, the better its drug loading efficiency. On the other hand, it may become unable to cross the corneal epithelium. Regarding the in vitro release study, the authors found that the optimal formulation released 63.6% of ofloxacin in 3 h compared to 99.55% of the commercial eye drops. A similar profile was obtained in the ex vivo permeation study, with a better performance of the optimized formulation and smaller particles, which can be achieved by increasing surfactant concentration. According to the group, nanoparticles adhere to the epithelium because of chitosan, but the accelerated permeation is due to PEG [41].

Unlike bacterial keratitis, the use of anti-fungal agents to treat fungal keratitis is quite challenging because of the poor water-solubility characteristic of these drugs. In order to overcome this and other problems, Roy et al. proposed the use of a microneedle ocular patch, which mimics the curvature of contact lenses, to improve the bioavailability of amphotericin B. To prepare the device, polydimethyl siloxane was used to produce the mold, and the patches were made of a combination of PVP (15% *w*/*v*) and PVA (15% *w*/*v*) that was powered into the molds. Afterward, the amphotericin B, previously loaded into liposomes with an encapsulation efficiency near 100%, was added to the polymer mixture in the convex mold and could be loaded up to 1.33% *w*/*w* within microneedles. Scanning electron microscopy (SEM) images revealed the liposomes on the surface of the microneedles. It was observed that the microneedles were completely dissolved within 60 s, and five minutes after the corneal insertion of microneedles in rabbits’ eyes, the baseplate of the patch was removed and the water content of the cornea could dissolve the PVP/PVA microneedles within minutes. The in vivo and ex vivo studies showed that the microneedle ocular patch containing amphotericin B was more effective compared to the liposomal amphotericin B-loaded microneedle ocular patch. Even though the drug distribution after administration did not encompass the entire eye globe, it was effective against *Candida albicans* infection [37].

Another group also proposed a new DDS to treat fungal keratitis with ecozanole, a strong and insoluble drug, to enhance the ocular permeation of eye drops. Li and collaborators (2018) fabricated eye drops made of carboxymethyl-α-cyclodextrin conjugated with chitosan, which were, at first, lyophilized with the drug to obtain a powder form, and then transformed into a suspension [50].

### 3.4. Application of Polymer Blends for Specific Ocular Tissues

#### 3.4.1. The Retina

The retinal pigment epithelium (RPE) is responsible for essential functions that enable vision, and its dysfunction is involved in some degenerative retinal and macular diseases. In this sense, biodegradable polymer blends have been used to facilitate RPE for transplantation, as reported by some authors.

When it comes to the need for the transplantation of RPE cells derived from pluripotent stem cells, polarized cells should be inserted under the retina, which limits manipulations and associates risks of iatrogenic lesions. Biomaterials emerge as a possibility to enhance retinal tissue formation and facilitate its integration in vivo. Among the numerous natural and synthetic polymers that can be used for this purpose, blending these compounds is also a great strategy to enhance the mechanical, degradable, and adhesion properties of the cell scaffolds. As reported by Hunt and collaborators (2018), in one study, HA was combined with low-viscosity methylcellulose to form an injectable hydrogel as a scaffold for the RPE and neural retina, which improved cell viability in vitro compared with controls [162].

According to Thomson et al., 2010, microcarriers have been investigated for mass cultivation and implantation of RPE cells. Moreover, they assessed the attachment of RPE cells to biodegradable PLLA (poly (lactic acid):PLGA microsphere blends for ocular transplantation. It was found that the characteristics of the microcarriers were affected by both the concentrations and blend ratios of the polymers used and that the addition of laminin to the surface of the blends could improve cell viability and bioresponsitivity [163]. Later, the same group tested different polymer blend ratios of PLLA and PLGA to evaluate their suitability as bio-scaffolds. The 25:75 (PLLA:PLGA) ratio blend showed the best results for the maintenance of cell proliferation and attachment throughout the study [164].

Retinal detachment is another serious disease that may cause visual dysfunction, and proliferative vitreoretinopathy is a common consequence. In this condition, vitrectomy is followed by tamponade with gas or silicone oil. Silicone oil is indicated in more severe cases, but it is not a biodegradable compound and must be removed through another surgery several months after the initial surgery. In addition, silicon oil impairs the solubility and release of some drugs. Cauldbeck et al. have proposed to minimize this issue by promoting a chain-end modification of polydimethylsiloxane with all-trans retinoic acid (an anti-proliferative and anti-scarring agent on RPE cells) and blending it with unmodified silicone oil. With this strategy, they were able to show a better solubility of the retinoic acid in the silicone oil, as well as a controlled release of the drug into the aqueous media [165]. Still, due to the innumerous complications that arise from the traditional use of silicone as mechanical support to the broken retina, absorbable buckles are now being tested as novel scleral-buckling materials. Chen et al. have demonstrated that a buckle made of chitosan-gelatin showed good mechanical properties and also supported the adhesion and growth of the human scleral fibroblasts, which makes this biodegradable blend a possible candidate for retinal detachment surgery [166].

In general, current treatments for posterior diseases include surgeries and some invasive techniques, such as intravitreal injections or implants. On the other hand, it is known that topical administration, in spite of being a non-invasive approach, has several limitations with respect to drug bioavailability and permeation through the biological barriers [167,168]. Nevertheless, an effort is being made to improve topical drug delivery with novel technologies, since it represents the major proportion of the market, in addition to having better patient compliance [9]. In a study conducted in 2018, the authors proposed eye drops with a PEGylated microemulsion containing dexamethasone to treat the posterior segment of the eye. Without any burst effect in the in vitro test, the entire loaded drug was released within 10 h. The formulation’s droplets were able to cross the cornea epithelium without damaging its tight junctions. The authors also compared the performance of the PEGylated microemulsion with a non-PEGylated one and, even though both of them could reach the rat’s retina via topical administration, the presence of PEG sustained higher levels of the drug at the site of action [169]. Tahara et al., 2017, managed to develop topical eye drops composed of surface-modified PLGA nanoparticles with chitosan, glycol chitosan, or polysorbate 80. Modified nanoparticles showed higher uptake into cells compared to the unmodified system, probably due to increased cell membrane fluidity. The in vivo time-course experiments revealed the increased intensity of the fluorescent nanoparticles after 30 min of topical instillation and, after 60 min, the nanoparticles had been cleared from the retina to the periocular circulatory systems. In general, surface modifications on the PLGA nanoparticles showed no signs of cytotoxicity, enhanced mucoadhesiveness because of the positive zeta potential, and better permeability between the anterior and posterior segments of the rat eye [170].

Diabetic retinopathy is another disease that impacts the retina, being the leading cause of sight loss in industrialized regions. It is mostly asymptomatic in the early stages and can be caused by structural and functional changes in the retinal vasculature, chronic exposure to hyperglycemia, and some other risk factors, such as hypertension [171,172]. The treatment of diabetic retinopathy is quite challenging due to invasive techniques, such as intravitreal injections. In a study conducted in 2021, the authors came up with bovine serum albumin (BSA) nanoparticles coated with HA, containing apatinib, a selective inhibitor of VEGF receptor 2, to be topically and intravitreally administered. In vitro studies revealed that increasing the BSA concentration led to a decrease in particle size, an increased positive zeta potential after coating with HA, and enhanced mucoadhesiveness. The system was proven to be well tolerated in retinal cells, and both coated and non-coated formulations presented a significant decline in retinal thickness with both administrations. Regarding the retinal distribution study, a highly significant increase in fluorescent intensity was observed for both formulations after intravitreal administration, whereas after topical instillation, only the coated nanoparticles could produce the same result [173]. In another study aiming at the treatment of diabetic retinopathy, Zeng and collaborators (2019) developed PLGA/chitosan nanoparticles containing interleukin-2, a cytokine with anti-angiogenic and antitumor efficacy. Despite the moderate drug encapsulation efficacy (34.7%), the formulation showed sustained drug release, with a strong anti-angiogenic effect and significantly lower cytokine expression after the intravitreal injection, compared to an interleukin-2 solution [174]. Similarly, Mahaling et al., 2018, evaluated nanoparticle eye drops consisting of a polycaprolactone core and Pluronic^®^ F68 shell, loaded with triamcinolone acetonide. The topical instillation of the eye drops could deliver the drug to the retina of diabetic retinopathy rats, which reduced the inflammation and improved the structural and functional activity of the retina. Moreover, according to the authors, this amphiphilic system could be used for the treatment of other diseases, such as diabetic macular edema, age-related macular degeneration, endophthalmitis, and retinitis pigmentosa [175].

#### 3.4.2. The Cornea

The corneal endothelium is the most crucial part of the cornea and its damage can cause corneal edema, opacity, and neovascularization, leading to severe impairment that may require corneal transplantation. However, transplantation depends on finding a donor and on the posterior non-rejection of the new tissue by the organism. These events make transplantation accessible, but limited. Like for the retina, tissue engineering using polymeric blends to develop new biomaterial matrixes has been the trend for corneal reconstruction (WANG et al., 2016). Endothelium replacement has taken an increasing place amongst cornea replacement strategies. According to Kong and Mi (2016), electrospinning has been largely used for fabricating biomimetic engineering functional corneal tissue. Electrospinning can provide support for cell adhesion, proliferation, and differentiation and also great mechanical properties. Yet, a blended electrospun scaffold, made by using a polymer blend, can regulate the mechanical, chemical, and biological properties of the scaffold (KONG and MI, 2016; TIWARI et al., 2016; YAO et al., 2017). In a study carried out by Hiep and Lee (2010), an electro-spun co-polymer PLGA/PCL blend, using PLGA 85:15, was used to fabricate fibrous mats. Considering that PCL is a flexible biopolymer used to overcome some limitations of PLGA, besides being relatively inexpensive, it was used as the main component. On the other hand, PLGA presents better cell adhesion and proliferation due to its hydrophilicity, contrary to the hydrophobicity of PCL. After testing different percentages of PLGA and PCL in the blend solution, they observed that the PLGA/PCL (20/80) showed the best mechanical strength, due to its uniform and small-diameter fibrous mat. Moreover, this system provided great fibroblast proliferation, indicating that the presence of PLGA in the mats improved biocompatibility, cell attachment, and proliferation (HIEP and LEE, 2010).

Young and collaborators (2014) investigated whether it was possible to create a blend made of chitosan and (PCL) to be used as a scaffold for corneal endothelial cell culturing and transplantation. As a result, they obtained transparent films with appropriate light transmittance when the concentration of PCL was raised to 30%; besides that, bovine corneal endothelial cells did not survive well in chitosan films, while on PCL hybridized into chitosan, the cells showed consistency and displayed a normal polygonal morphology. Moreover, the biodegradation of the chitosan/PCL membranes was optimized to a controllable rate, which suggests that this system may provide bioengineered corneal endothelia in the future [176]. Similar results were obtained by Wang et al., 2019, who also demonstrated the same system (chitosan/PCL 25%) as a suitable alternative for cornea transplantation. However, disadvantages such as reduced ability to support epithelial cells after long-term co-culture due to limited biodegradability and possible adverse tissue response are cited by the authors as current challenges of artificial substrates [177]. In a previous study by the same research group, they evaluated the cultivation of corneal endothelial cells in different biomaterials. They showed that these cells did not attach to PVA surfaces, but showed initiated adhesion to poly (ethylene-co-vinyl alcohol) (EVAL), tissue culture polystyrene (TCPS), and polyvinylidene (PVDF). As a background result for the following experiments, they have shown the great potential of the novel chitosan/PCL blended biomaterial for the transplantation of bioengineered corneal endothelium.

## 4. Final Considerations

The use of polymers in the development of drug delivery systems is widespread and well-established. Although technological development has allowed significant modifications in the chemical structure of polymers and even the synthesis of new compounds, the association of two or more polymers, so-called polymer blends, offers numerous advantages. The main features are the possibility of modulating the physicochemical, mechanical, and biological properties of the system, besides the great cost–benefit ratio.

Since ocular tissues are complex and the entire anatomical structure of the eye seeks to protect tissues from systemic circulation and external agents, the use of materials such as polymers as the main component of DDS may be challenging in issues of toxicity and biocompatibility aligned to the performance of the proposed system. In fact, it is precisely the difficulty in finding materials that meet all the requirements as a viable release system that leads to the search for alternatives. The association of polymers occurs solely in a physical form, without chemical reactions, leading to a gain in properties compared to pure materials. In the works presented in this article, different gains with the use of blends are reported, from the increased efficiency of drug encapsulation to greater viability and cell adhesion, not to mention a better modulation of drug release rate.

The search for “polymer blends” in the Pubmed and Scopus databases generates numerous results; however, they correspond to only approximately 2% of the number of results obtained by searching for “polymers”. When the search is for “polymer blends for ophthalmic use” (or other corresponding terms), the number of results is usually fewer than 20 papers.

Interestingly, considering the search conducted in this paper for the works in which more than one polymer was used, none of them used the term “blend” or “polymer blend” in the title, and just a few mentioned it in the text. This may be an indication of the still-low compliance to the use of the blending concept, even though, in practice, it is applied; nevertheless, in all papers in which a blend was used, the authors emphasized the performance advantages of using a combination of polymers as a DDS.

## 5. Conclusions and Future Perspectives

Based on the evidence discussed in published studies, there is increasing interest in betting on the use of polymer blends, as already described, as well as in the search for new polymer associations. The versatility of these combinations emerges as a technology proposal capable of optimizing and innovating the applicability of DDS for the treatment of ocular diseases. Yet, regulatory pathways for further clinical applications of these new polymers’ associations remain to be determined.

## Figures and Tables

**Table 1:** List of papers regarding polymer blends found from the search for polymeric drug delivery systems for ophthalmic use in the databases Pubmed and Scopus.

Author	Target	Drugs	Polymers	Technology	Administration
Anterior Ocular Disorders					
[32]	Corneal wound healing	Ferulic acid	Pluronic^®^ F68 and hyaluronan	Nanocomposite (micelle-nanogel)	Topical (ocular)
[33]	Infectious ocular keratitis	hLF 1-11 (synthetic antimicrobial peptide derived from human lactoferrin)	Hydroxypropylmethylcellulose (HPMC) and HA	Freeze-dried ocular insert	Topical (ocular)—no in vivo tests
[34]	Subconjunctival retention	Sunitinib malate	Methylcellulose (MC), HA and PLGA	Microparticles	Subconjunctival injection
[35]	Ocular hypertension	Dorzolamide HCl	Chitosan, PCL, and PVA	Polymeric nanoparticles	Topical (ocular)
[36]	Cornal permeability	Myricetin	Polyvinyl caprolactam, polyvinyl acetate, and polyethylene glycol graft copolymer	Polymeric micelles	Topical (ocular)
[37]	Fungal keratitis	Amphotericin B	PVA and PVP	Microneedle ocular patch (polymer composite)	Micromolding technique to mimic contact lenses
[38]	Steroid-induced cataract	Triamcinolone acetonide	PLC and Pluronic^®^ F68	Polymeric core-shell nanoparticles	Topical (eye drops)
[39]	Ocular hypertension	Timolol maleate	HPMC and HA	Composite ocular films	Topical (eye drops)
[40]	Increase hydrophobic drugs penetration	Tacrolimus	Amino-terminated poly(ethylene glycol-block-poly(D,L)-lactic acid) (NH2-PEG-b-PLA) and HPMC	Nanomicelles	Topical (eye drops)
[41]	Bacterial Keratitis	Ofloxacin	Chitosan and PEG	Enhanced lipid nanoparticles	Topical (eye drops)
[42]	Corneal neovascularization	Axitinib	MPEG and PCL	Polymeric micelles	Topical (ocular)
[43]	Ocular hypertension	Dorzolamide HCl	Carbopol and HPCM	pH-triggered in situ gel (ISG)	Topical (ocular)
[44]	Dry eye disorders and corneal ulcer	Levofloxacin	HPMC and sodium alginate	pH-triggered in situ gel (ISG)	Topical (ocular)
[45]	Ocular drug delivery	Azelastine HCl	Pluronic^®^ F127 and carbopol	Polymeric micellar gel	Topical (eye drops)
[46]	Ocular drug delivery	Levofloxacin	Eudragit^®^ RS and carbopol	Mucoadhesive minitablets	Topical (ocular)
[47]	Ocular drug delivery	Triamcinolone acetonide	α,β-poly(N-2-hydroxyethyl)-D,L-aspartamide (PHEA), and poly-butylene succinate (PBS)	Microfibrillar polymeric ocular inserts	Topical (ocular)
[48]	Ocular drug delivery	Small peptides	Polyoxyethylene hydrogenated castor oil 40 (HCO-40) and octoxynol 40 (OC-40)	Self-assembling multi-layered nanomicelles	Topical (ocular)
[49]	Glaucoma	Timolol maleate	Chitosan, PVP, and poly (N-isopropylacrylamide)	Ocular contact lenses	Topical (ocular)
[50]	Fungal keratitis	Econazole	Carboxymethyl-α-cyclodextrin and chitosan	Eye drops	Topical (ocular)
[51]	Anterior segment of the eye	Betaxol hydrochloride	Cellulose acetate and Eudragit S100	Inner layer-embedded contact lenses	Topical (ocular)
[52]	Anterior segment of the eye	Diclofenac sodium	Ethyl cellulose and Eudragit S100	Inner layer-embedded contact lenses	Topical (ocular)
[53]	Controlled release of poorly bioavailable drugs into the aqueous humor	Cannabigerolic acid	Hydrogel: Methylcellulose (MC) and HAnanoparticles: poly(ethylene oxide) and PLA	In-situ forming nanoparticle-laden hydrogel	Topical (ocular)
[54]	Ocular hypertension	Timolol maleate	Chitosan and gelatin	Hydrogel	Topical (ocular)
[55]	Bacterial growth	Moxifloxacin hydrochloride, chlorhexidine diacetate monohydrate, and diclofenac sodium salt	Sodium alginate, HA, chitosan, and polylysine hydrobromide	Layer-by-layer coatings on contact lenses (hydrogel)	Topical (ocular)
[56]	Dry eye syndrome	Cyclosporine A	PEG and PLA	Polymeric micelles	Topical (ocular)
[57]	Glaucoma	Timolol (precursor)	PEG and polyamidoamine (PAMAM)	Polymeric dendrimer	Topical (ocular)
[58]	Autoimmune uveitis	Cyclosporine A	Methoxy-poly(ethylene-glycol)-hexyl substituted poly-(lactic acid) (mPEGhexPLA)	Nanocarriers	Topical (ocular)
[59]	Keratoprosthesis, orthokeratology, and mini-scleral lens	-	Ester-based polyurethane (EBPU), N,N-dimethylacrylamide (NNDMA), N-vinyl pyrrolidone (NVP), and acryloylmorpholine (AMO)	High modulus hydrogels	Topical (ocular)
[60]	Hyperacute bacterial conjunctivitis and endophthalmitis	Tobramycin sulfate	Chitosan HCl and Poloxamer 407	Mucoadhesive microparticles incorporated in thermosensitive in situ gel	Topical (ocular)
[61]	Glaucoma	Acetazolamide	HA and PEG	Polymeric films	Topical (ocular)
[62]	Corneal keratitis or bacterial endophthalmitis	Moxifloxacin	HPMC, PVP-K30, and PEG	Ocular inserts	Topical (ocular)
[63]	Bacterial keratitis	Ceftazidime	Chitosan, HPMC, and HA	Mucoadhesive nanoparticles	Topical (eye drops)
[64]	Corneal delivery	Besifloxacin	PVA and PVP	Polymeric microneedles	Topical (ocular)
[65]	Anterior segment of the eye	Curcumin	PVCL, PVA, and PEG	Polymeric nanomicelles	Topical (ocular)
[66]	Glaucoma	Resveratrol	PEG and chitosan	Polymeric nanoparticles	Topical (ocular)
[67]	Glaucoma	-	Poly(N-isopropylacrylamide) and gelatin	Hydrogel	Intracameral injection
[68]	Dry eye syndrome	Epigallocatechin gallate	Gelatin-gpoly(N-isopropylacrylamide)	In situ gelling carriers	Topical (ocular)
[69]	Fungal keratitis	Amphotericin B	Chitosan and Poloxamer^®^ 188	Nanostructured lipid carriers	Topical (ocular)
[70]	Glaucoma	Acetazolamide	Ethyl cellulose and Eudragit RS100	Polymeric nanocapsules	Topical (ocular)
[71]	Glaucoma	Pilocarpine hydrochloride	Gelatin-gpoly(N-isopropylacrylamide)	In situ forming hydrogel	Intracameral injection
**Posterior ocular disorders**					
[72]	Retinal diseases	Erythropoietin	Chitosan and hyaluronic acid (HA)	Nanoparticles	Topical (ocular)
[73]	Systemic absorption and brain-targeting effect	Vinpocetine	Carbopol and HPCM	pH-triggered in situ gel (ISG)	Topical (ocular)
[74]	Treatment of mid-posterior diseases.	Glycylsarcosine	Chitosan-glutathione	Functional intercalated nanocomposites	Topical (ocular)
[75]	Proliferative vitreoretinopathy	Ibuprofen and all-trans retinoic acid	Dimethylsiloxiane, PEG, and silicone	Polymer grafts	-
**General ocular disorders**					
[76]	Delivery to ocular tissues	-	Polysaccharides	In situ forming gel	Topical (ocular)
[77]	Ocular drug delivery	-	-	Liposomes, SLN, NLC, niosomes...	Ocular
[78]	Ocular drug delivery	-	-	Contact lenses	Topical (ocular)
[79]	Ocular drug delivery	-	-	Polymeric nanomicelles	Topical (ocular)
[80]	Ocular drug delivery	-	-	Contact lenses	Topical (ocular)
[81]	Improve drug delivery and encapsulation in nanocarriers	-	Chitosan, PLGA, alginate	Nanoparticles	Topical (ocular)
[82]	Improve drug delivery and residence time	-	Gellan gum and Pullulan	Electrospun nanofibers	Topical (ocular)
[83]	Bacteria and fungi ocular infections	-	Two antibacterial synthetic polymers with dipyridine motif	?	Topical (ocular)
[3]	Ocular drug/gene delivery	-	PLGA, chitosan and gelatin	Nanocarriers	Ocular
[14]	Ocular drug delivery	-	-	Micro and nanoparticles (gels)	Topical (ocular)
[84]	Drug delivery	Triamcinolone acetonide and ovoalbumin	PEG and diacrylate (PEGDA)	Implants	-
[85]	Oncology and ophthalmology	-	-	Molecularly imprinted polymers (MIP)	-
[86]	Ocular inflammation	Tacrolimus	PEG_2000_ and derivatives	Polymeric micelles	Topical (ocular)
[87]	Ocular drug delivery	Dexamethasone sodium phosphate	Poloxamer 188 and Poloxamer 407	In situ gel loaded with nanoparticles	Topical (ocular)
[88]	Sustained delivery of macromolecules	Insulin, catalase, octreotide, IgG, IgG Fab, Lysozyme, BSA	P(CL-co-GA)-PEG-P(GA-co-CL)	Polymeric nanoparticles	Ocular
[89]	Neovascular ocular diseases	Imatinib	HA and PEG	Polymeric micelles	Topical (ocular)
[90]	Ocular drug delivery	Infliximab	HA, N-isopropylacylamide (pNIPAAM) and PEG	Collapsible hyaluronic acid hydrogels	Intraocular injection
[91]	Ocular inflammatory disorders	Pioglitazone	PLGA and PEG	Polymeric nanospheres	Topical (ocular)
[92]	Ocular drug delivery	Timolol maleate, dexamethasone, and dorzolamide hydrochloride	Poloxamer 407, Poloxamer 188, and chitosan	In situ forming ophthalmic gel	Topical (eye drops)
[93]	Ocular drug delivery	Pilocarpine hydrochloride	Cellulose and Poloxamer 407	In situ gelling thermo-responsive hydrogels	Topical (ocular)
[94]	Intraocular inflammation	Moxifloxacin hydrochloride	Chitosan and HA	Lipid-polymer hybrid nanoparticles	Topical (ocular)
[95]	Ocular drug delivery	-	-	Polymeric micelles	Ocular
[96]	Ocular drug delivery	-	-	Lens-based and conventional drug delivery	Topical (ocular)
[97]	Choroidal neovascularization	Cell-penetrating peptides	PEG and PLGA	Polymeric nanoparticles	Topical (ocular)
[98]	Ocular drug delivery	Vancomycin	Eudragit^®^ RS100 and carbopol	Polymeric nanoparticles	Topical (ocular)
[99]	Ocular drug delivery	Pilocarpine hydrochloride	Poloxamer 407 and gellan gum	In situ gelling systems	Topical (ocular)
[100]	Ocular drug delivery	Flurbiprofen	PCL and poloxamer 188	Freeze-dried polymeric nanoparticles	Topical (eye drops)
[101]	Delivery to ocular tissues	-	-	Contact lenses	Topical (ocular)
[102]	Ocular drug delivery	-	-	Dendrimers	-
[103]	Ocular drug delivery	-	Elastin-like polypeptides	-	-
[104]	Ocular inflammation and infection	Dexamethasone sodium phosphate and Tobramycin sulfate	Poloxamer 407 and HPMC K4M	Thermoresponsive ophthalmic in situ gel	Topical (ocular)
[105]	Ocular drug delivery	Fluorescein sodium and ofloxacin	Pluronic^®^ F127 and Pluronic^®^ F68	In situ gelling system	Topical (ocular)
[106]	Ocular drug delivery	Lidocaine	PLGA and collagen	Polymeric nanoparticles	Topical (ocular)
[107]	Ocular drug delivery	Diclofenac sodium	Methoxy PEG-PCL copolymers and α-cyclodextrin	Micellar supramolecular hydrogel	Topical (ocular)
[108]	Ocular drug delivery	-	-	Polymeric microsponges	Topical (ocular)
[109]	Ophthalmic gene therapy	-	-	-	-

**Table 2:** Main diseases and/or tissues addressed and the respective polymers used in the proposed delivery systems.

Disease/Tissue	DDS (Technology)	Most Used Polymers
Posterior segment of the eye	Nanoparticles/nanocomposites/polymer grafts	Chitosan/HA/PEG
Anterior segment of the eye	Micelles/contact lenses	PCL/PEG/PVA/PVP/EC/Eudragit^®^
Keratitis	Inserts/composites/nanoparticles	HPMC/HA/PVA/PVP/chitosan/PLGA
Dry eye	In situ hydrogel/micelles	HPMC/PEG/gelatin
Glaucoma	Nanoparticles/in situ hydrogel	Chitosan/HPMC/HA/PEG/gelatin
DDS	In situ hydrogel/nanoparticles	Poloxamers/PLGA/PEG/carbopol
